# Cognitive Biases and Emotional Symptomatology as Predictors of Changes in Peer Victimization: A Longitudinal Structural Equation Modeling Study

**DOI:** 10.1007/s10802-025-01337-7

**Published:** 2025-06-02

**Authors:** Fabiola Espinosa, Ivan Blanco, Nuria Martin-Romero, Alvaro Sanchez-Lopez

**Affiliations:** 1 Department of Personality, Evaluation and Clinical Psychology, Faculty of Psychology, Complutense University of Madrid, Campus de Somosaguas, Pozuelo de Alarcón, Madrid, 28223 Spain; 2 Department of Educational Sciences, University of Alcala, Alcala de Henares, Spain

**Keywords:** Bullying, Peer victimization, Cognitive biases, Depression, Path analyses, SEM

## Abstract

Bullying and peer victimization are major public health concerns among adolescents. In recent years, a bidirectional relationship between emotional symptoms and peer victimization has been proposed. Cognitive theories emphasize the role of cognitive biases (i.e., attention and interpretation) in the onset and maintenance of emotional symptoms. Considering this framework, this study aimed to examine whether cognitive biases predict temporal changes in peer victimization (i.e., physical, verbal-relational, and cyberbullying) by contributing to higher levels of emotional symptomatology. A sample of 179 adolescents aged 13–16 years old (*M* = 14.5) was assessed at two time points by completing self-report measures of peer victimization (CMIE-IV), depressive and anxious symptoms (SMFQ and GAD, respectively), and an experimental task (SST) for the assessment of cognitive biases of attention and interpretation. Structural equation modeling was conducted to examine whether emotional symptoms at T2 mediated the relationship between cognitive biases at T1 and changes in different forms of peer victimization from T1 to T2 (i.e., three months). Results showed an indirect effect of attention bias on changes in different types of peer victimization through interpretation bias and depressive symptoms. Likewise, attention bias had a direct effect on interpretation bias, and interpretation bias had a direct effect on depressive symptoms. These findings support the central role of cognitive biases in understanding the occurrence of depressive symptomatology and its potential impact on adolescents’ higher risk of suffering from different peer victimization experiences.

**Supplementary Information:**

The online version contains supplementary material available at 10.1007/s10802-025-01337-7.

## Introduction

### Peer Victimization During Adolescence

Bullying is considered a public health problem among adolescence (Ford et al., 2017; Moore & Woodcock, 2017) given its impact on mental health, academic performance and social development (Halliday et al., 2021; Polanin et al., 2021; Ragusa et al., 2023; Wang et al., 2024). Bullying arises when one or more adolescents repeatedly use violence against another, involving an imbalance of power between the bully and the bullied (Olweus, 1994). Bullying violence can be physical (e.g., hitting, pushing), verbal-relational (e.g., insults, spreading rumors), or take forms of cyberbullying (e.g., threatening, doxing) using electronic media such as mobile phones or social networks for carrying out the aggression (Olweus, 1993; Smith, 2014). According to data from two large-scale international surveys – the Global School-based Student Health Survey (GSHS), and the Health Behaviour in School-aged Children (HBSC), one out of three students experience some form of peer victimization due to bullying during adolescence (UNESCO, 2019). Besides the physical and academic damage caused by bullying, adolescents who experience peer victimization are more likely to also experience psychological distress and emotional problems. Studies indicate that being bullied is associated with increased feelings of loneliness, sleep problems, a higher likelihood of engaging in risky sexual behaviors, and an increased risk of dropping out of school (UNESCO, 2018). Additionally, victims of bullying tend to have higher drug use (Airagnes et al., 2023) and greater risk for suicidal ideation and the development of emotional symptomatology (Halliday et al., 2021; Moore et al., 2017). Further, these negative effects seem to persist in adulthood (Arseneault, 2018). Due to these consequences, identifying the risk and protective factors is highly relevant for the prevention and early intervention of peer victimization during adolescence.

### Emotional Symptomatology and Peer Victimization

Depression and anxiety in adolescents are also a highly prevalent and serious public health problem (Patel et al., 2007). Importantly, in recent years, depression and anxiety have been recognized not only as consequences of peer victimization (Halliday et al., 2021; Moore et al., 2017), but also as strong risk factors for experiencing it (Christina et al., 2021; Reijntjes et al., 2010). In this sense, some authors (Forbes et al., 2019; Klomek et al., 2019) have examined the specific contributions of emotional symptomatology to predict different types of peer victimization. Klomek et al. (2019) investigated bidirectional associations between depression and various forms of victimization (relational, verbal, and physical) in adolescents aged 13–18 years. They found that adolescents with higher depressive symptoms at baseline were more likely to subsequently experience more relational, physical, and verbal victimization at three months. Furthermore, more depression symptoms at three months predicted a higher likelihood of experiencing relational and verbal victimization at 12 months, but not physical victimization. Similarly, Forbes et al. (2019) evaluated bidirectional associations between symptoms of anxiety and depression, and physical, verbal-relational, and cyber victimization. They found that higher levels of depressive symptoms in adolescents (aged 10–11) predicted a higher likelihood of experiencing physical and relational peer victimization two years later. Regarding cyberbullying victimization, anxiety emerged as the primary indicator in this study. These findings highlight how the experience of emotional symptoms (such as depression and anxiety) can contribute to the occurrence of peer victimization. Furthermore, there may be specific relationships between the type of bullying experienced and the emotional symptoms that predict those peer victimization processes.

These findings can be aligned with the predictions of the vulnerability-stress models from a developmental perspective (see Gibb & Coles, 2005). According to these models, the stress caused by bullying experiences in adolescence may reinforce and sustain emotional symptomatology, which could help explain the ongoing cycle of peer victimization. In this respect, some authors have suggested a specific link between peer victimization and depressive symptoms during adolescence due to the development of a cognitive style within the context of bullying that reinforces negative evaluations about oneself and the future (e.g., I do everything wrong, I am worthless, my life will always be horrible, etc.) - (see Wang, 2011). Similarly, a link between anxiety symptoms and peer victimization across development could be explained by the impact of bullying, which produce sustained states of alertness, hypervigilance and a higher tendency to overestimate threats in everyday situations (see Espelage & Holt, 2001; Roth et al., 2002). Moreover, other authors have proposed that observable symptoms of depression or anxiety that some adolescents may exhibit (e.g. isolation, fear, crying, flat affect) may be interpreted by bullies as signs of weakness and vulnerability, increasing their aggression towards these adolescents (Luchetti & Rapee, 2014; Schacter & Juvonen, 2017). Additionally, it has also been suggested that adolescents with emotional problems may have difficulties defending themselves in such situations (Reijntjes et al., 2010), so bullies might see them as easy targets. Taken together, emotional symptomatology may not only be a consequence of peer victimization but may also contribute to its occurrence and development. From this point of view, studying the mechanisms that predict the risk of experiencing emotional symptomatology during adolescence might also be informative in identifying the pathways through which processes of peer victimization may evolve across this crucial developmental period.

### Cognitive Biases and Emotional Symptomatology

Cognitive biases such as attention (i.e., the tendency to preferentially process emotional information in a specific way, giving more attention to negative or positive information; Armstrong & Olatunji, 2012) and/or interpretation biases (i.e., the tendency to preferentially interpret emotionally ambiguous information in a specific way, interpreting information positively or negatively; Clark et al., 2000) have been both considered main mechanisms for the onset and maintenance of emotional symptomatology (Gotlib & Joorman, 2010; Joormann & Vanderlind, 2014). According to cognitive theories of emotional disorders, individuals who are more prone to experience depressive and anxious symptomatology have negative schemas that can be triggered by stressful situations (e.g., bullying experiences). Activating these negative schemas would then direct the information processing towards negative information (i.e., negative cognitive biases activation) - (Beck, 1976; Williams et al., 1997). Importantly, these biases seem to operate co-jointly. The Combined Cognitive Bias Theory (e.g., Everaert et al., 2014) states that attention would be the main entry mechanism for emotional information, where attended internal and/or external information is then gathered for subsequent processing (i.e., interpretation of gathered information). Thus, negative attention bias leads to negative interpretation biases. Empirical evidence supports this idea. For instance, Everaert et al. (2014) showed that, in young adults with subclinical depression, a stronger negative attention bias predicts a stronger negative interpretation bias. Similarly, Sfärlea et al. (2021) found a significant association between attention and interpretation biases in adolescents. Considering these findings, interpretation bias might partially mediate the effects of attention bias on depressive symptomatology during adolescence.

Previous research on adolescents suggests that cognitive biases play a key role in the onset and persistence of emotional symptoms during this stage of development (see Platt et al., 2017; Stuijfzand et al., 2018). Specifically, a positive relationship has been found between negative attention and interpretation biases and both anxiety (Klein et al., 2018; Smith et al., 2018) and depressive symptoms (Orchard & Reynolds, 2018; Osinsky et al., 2012; Platt et al., 2023). Overall, negative biases in attention and interpretation likely contribute to higher levels of emotional symptomatology during adolescence. In turn, these symptoms might directly raise the risk of experiencing peer victimization at this stage of development. However, this set of temporal relationships has yet to be tested.

### The Present Study

In line with previous research supporting the association between cognitive biases and emotional symptomatology in adolescents, (Platt et al., 2017; Stuijfzand et al., 2018) as well as, considering recent evidence on the bidirectional relationships between emotional symptomatology and different forms of bullying peer victimization (Christina et al., 2021), the aim of the present study was to test the predictive role of cognitive biases (negative attention and interpretation biases) in accounting for time changes in different types of peer victimization (physical, verbal-relational, and cyberbullying), considering emotional symptomatology (symptoms of depression and anxiety) as the mediators of these relationships. Specifically, we first hypothesized that there would be a positive association between negative attention and interpretation biases, as it has been found in young adults by Everaert et al. (2014) and in adolescents by Sfärlea et al. (2021). Second, we expected that a direct effect of cognitive biases (both attention and interpretation) on emotional symptomatology would emerge in accordance with cognitive theories (Beck, 1976; Williams et al., 1997) and recent empirical research supporting those associations (Platt et al., 2017; Stuijfzand et al., 2018). Third, we expected that there would be a direct effect of emotional symptomatology in predicting temporal changes in the levels of different types of peer victimization across time (see Christina et al., 2021). Finally, we examined whether cognitive biases indirectly predict changes in peer victimization over time. Specifically, we examined whether higher levels of emotional symptoms mediate the relationship between cognitive biases and different types of peer victimization (i.e., a specific model considering the temporal path of negative attention bias at T1 ◊ negative interpretation bias at T1 ◊ emotional symptoms at T2 ◊ changes from T1 to T2 in separate forms of peer victimization).

## Method

### Participants

A total of 291 adolescents participated in the study. The sample was recruited from two public secondary schools in Madrid, Spain. These schools were randomly selected from a list of public secondary schools and were contacted sequentially until the required sample size was reached. The following criteria were used to determine participant eligibility for the study: age between 13 and 17 years, adequate comprehension of the language used in the assessments, and signed consent from the participant and their parents or legal guardians. The informed consent document thoroughly explained the study and guaranteed data anonymity. Prior to the start of the study, this document was provided to and signed by both parents/legal guardians and adolescents. No financial or material compensation was offered for participation.

Participants completed self-report measures of emotional symptoms, peer victimization and an experimental task assessing cognitive biases at baseline (i.e., Time 1; T1). The experimental task was applied using a computerized version on participants’ phones (see full details below). From the original pool of participants, 112 participants (38% of the sample) could not complete the study because they were unable to complete the experimental task (i.e., due to missing their phones at the time of the evaluation, phone-related technological issues, or because they did not correctly follow the task instructions during the completion). Thus, the final sample in the study comprised 179 participants (54% females; 46% males) aged 13 to 16 years (*M* = 14.5, *SD* = 0.70) of whom 94.4% were of Spanish nationality. This sample size is comparable to previous studies that have tested the relationship between individual differences in cognitive biases and emotional processes during adolescence (see, for instance, Platt et al., 2023; Sfärlea et al., 2021). This sample had valid data for the negative attention and interpretation bias measures at the beginning of the study and completed the self-report measures both at baseline and again in a follow-up, three months later (i.e., Time 2; T2; see full details in the Procedure section, below). No attrition issues were observed in the study (see Supplementary for details on comparisons between completers and non-completers of the cognitive task in the rest of main variables assessed).

The study was approved by the Ethics Committee of Complutense University of Madrid (reference number: CE_20210120-18_SOC) and followed all the guidelines of the Declaration of Helsinki (WMA, 2013).

### Measures

*Peer Victimization*: The assessment of different types of peer victimization experienced was carried out by adapting the Multimodal School Interaction Questionnaire (CMIE-IV; Caballo et al., 2012) for its use in the current study. This self-reported questionnaire in its original form consists of 36 items and can be applied from 10 years old to older. It is distributed across five factors: (a) Intimidating behavior (bully), (b) Victimization received (bullied), (c) Active observer in defense of the bullied, (d) Extreme bullying/ Cyberbullying, and (e) Passive observer. For the purpose of this study, we only used the factor of peer victimization received, which includes information on the different types of peer victimization considered in the current study (i.e., physical, verbal-relational, cyberbullying). Due to the absence of certain bullying behaviors, not covered by the original items of the questionnaire (e.g., lack of assessment of some forms of current cyberbullying victimization in social networks such as Instagram or Tik Tok), we decided to update the assessment instrument adding a small number of new ad-hoc items to fully address relevant experiences for each type of victimization. These new items included one for physical victimization–“*My belongings have been taken*,* damaged*,* or stolen without my permission*”, one item for verbal-relational victimization – “*They have tried to turn my classmates against me*”, and two items for cyberbullying victimization – “*They ignore me (ignore my messages*,* friend requests*,* etc.) on mobile and r online platforms (WhatsApp*,* TikTok*,* Twitter*,* Instagram…)*” and “*They have shared sexual images or videos of me on the internet or passed them to others via their mobile phone without my consent*”). According to the information on the use of the original form of this questionnaire, indices of the levels of the different types of victimization can be obtained by selecting the above-mentioned items (Caballo et al., 2012). However, to verify that including the new ad-hoc items to those separate victimization factors maintained the structure given by the original instrument, a confirmatory factor analysis was performed for each type of victimization (i.e., physical, verbal-relational, and cyberbullying) following a structural after measurement approach (Rosseel & Loh, 2024) (see Supplementary Material for more details). These analyses confirmed that, after the incorporation of the new ad-hoc items, the final received global factor of peer victimization was distributed, into the following three separate sub-factors: five items related to physical victimization (e.g., “*My belongings have been taken*,* damaged*,* or stolen without my permission*”), eight items related to verbal-relational victimization (e.g., “*I have been insulted*”), and five items related to cyberbullying victimization (e.g., “*I have received threats*,* insults or negative comments via the internet*,* mobile phone*,* social networks (WhatsApp*,* TikTok*,* Instagram*,* Twitter…)*, etc.“). Response options for each item included four choices (1: never; 2: few times; 3: several times; 4: many times). The internal consistency reflected by the global factor of bullying victimization experienced in the present study was good (T1: *α* = 0.86; T2: *α* = 0.89). In line with the objectives of this study, internal consistency was also calculated for each subtype of bullying victimization separately in each time of evaluation (physical bullying: T1: *α* = 0.60, T2: *α* = 0.69; verbal-relational bullying: T1: *α* = 0.79, T2: *α* = 0.84; cyberbullying: T1: *α* = 0.59, T2: *α* = 0.64).

*Depressive symptoms*: The assessment of depressive symptoms was conducted using The Short Mood and Feelings Questionnaire (SMFQ; Angold & Costello, 1987). This self-reported questionnaire consists of 13 items applicable from eight years of age. Responses are rated on a Likert scale with three response options (0: not true; 1: sometimes; 2: true). In the current study, internal consistency was excellent (T1: *α* = 0.91; T2: *α* = 0.94).

*Anxiety symptoms*: The Generalized Anxiety Disorder 7-item scale (GAD-7; Spitzer et al., 2006) was employed to assess the presence and severity of anxiety symptoms. This self-reported scale comprises seven items, offering four response options (0: not at all; 1: several days; 2: more than half the days; 3: nearly every day). Previous validation studies (e.g., Crockett et al., 2022; Pérez-Pedrogo et al., 2022) conducted in adolescents aged 12–17 years have demonstrated that the GAD-7 exhibits acceptable specificity and sensitivity in identifying clinically significant anxiety symptoms. In the present sample the internal consistency was excellent (T1: α = 0.90; T2: α = 0.93).

*Cognitive Bias*: A computerized version of the Scrambled Sentences Test (i.e., SST; Wenzlaff & Bates, 1998) was used for the assessment of attention and interpretation biases at T1. Participants performed the task, using a web-based app version of the computerized version of the SST, being completed on their phones. This version of the task, runnable on cellphones has been previously used to establish reliable indicators of cognitive biases related to emotional symptomatology in response to stressors (Blanco et al., 2023).

The aim of this task was to form grammatically correct sentences using five out of the six available words (e.g., *of proud are others ashamed of me*). Each sentence could be constructed in either positive (e.g., *others are proud of me*) or negative (e.g., *others are ashamed of me*) manner. The main task consisted of ten trials with grammatically scrambled emotional sentences. The number of trials was determined based on extensive prior research (Martin-Romero & Sanchez-Lopez, 2023; Blanco et al., 2023), which assessed the minimum SST trials necessary to obtain reliable interpretation bias indices related to individual differences in stress and depression levels. Moreover, additional piloting was conducted to determine a format sufficient for obtaining reliable indicators of attention and interpretation biases in comparable adolescent samples.

**Fig. 1:**
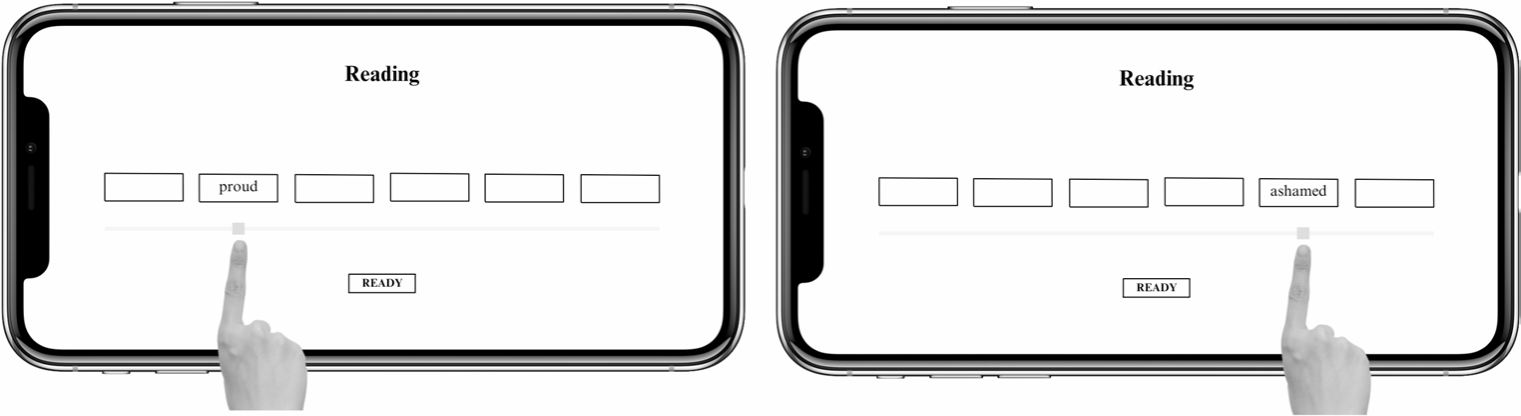
Example of the reading phase

**Fig. 2:**
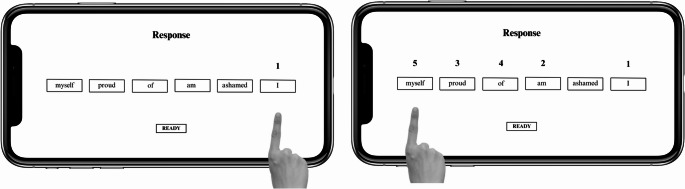
Example of the response phase

Prior to completing the task, participants performed ten test trials with neutral sentences, as an initial practice phase, to get familiar with the procedure of the task. Each trial had two distinct phases: a reading phase and a response phase. The task began with the presentation of a fixation cross on the left side on the phone screen. Once the participants pressed on the fixation, the reading phase began. During the reading phase, six blank boxes appeared across the center of the screen, each containing a hidden word from the scrambled sentence. To reveal a word, participants slid their finger along a scale beneath each box. Only the word in the selected box was displayed, while the others remained blank (see Fig. 1). Participants had a maximum of 14 s to view each word and mentally form a grammatically correct sentence. Once the reading time was finished, or if the participant clicked on the “ready” option on the screen, the response phase began. During this phase, the previously hidden words were displayed on the screen. Participants had to reconstruct the grammatically correct sentence they had mentally unscrambled in the reading phase. To do this, they tapped each word in their chosen order, with numbers (1–5) appearing above the words in the sequence they were selected (Fig. 2). This phase had a set time limit of four seconds.

During the reading phase, the task captures the time participants spend attending to the positive (*proud)* and negative (*ashamed*) words. A negative attention bias index is calculated by dividing the time attending to the negative word by the time attending to both positive and negative words across the trials. The attentional bias index ranged from 0 to 1, with 0.5 indicating no bias, values below 0.5 reflecting a positive bias, and values above 0.5 indicating a negative bias. Additionally, a negative interpretation bias index was calculated as the proportion of negatively coded (i.e., unscrambled) sentences relative to the total number of correctly completed emotional sentences. Similar to the attention bias index, the interpretation bias index ranged from 0 to 1, where higher values indicate greater negative interpretation bias, lower values indicate greater positive interpretation bias, and 0.5 represents no bias.

As mentioned above, this task has been widely used and previously validated (see Blanco et al., 2021; Blanco et al., 2023; Boemo et al., 2024; Everaert et al., 2014; Sanchez et al., 2015; Würtz et al., 2022) and additional piloting was conducted in adolescent samples before starting the collection data of the current study.

### Procedure

Data collection was carried out at two different time points in the classrooms, under the supervision of several researchers. Prior to data collection, adolescents and their parents or legal guardians received an informed consent document explaining their participation in the study and ensuring data anonymity. Once the parents and the adolescents signed the informed consent forms, the study began with an initial session lasting approximately one hour. In this session, adolescents completed the SST task on their phones and the psychological self-report questionnaires on paper-and-pencil formats. The second session took place three months later and lasted approximately half an hour. During this session, participants completed the follow-up psychological questionnaires and were then fully debriefed about the study’s purpose.

### Statistical Analysis

As preliminary steps, prior to the main analysis, a simple imputation of missing values was first performed using the Expectation-Maximization (EM) method on the psychological scales (i.e. symptomatology, bullying). After imputation, the validity of the data was checked through sensitivity analysis. Descriptive analyses were then conducted for each study variable. Furthermore, standardized residuals of change from T1 to T2 were calculated for the bullying victimization variables (i.e., physical, verbal-relational, and cyberbullying victimization). Using standardized residuals provides a reliable measure of change over time while controlling for variability among differences in baseline scores between participants (see, for instance, Segal et al., 2006).

Tests of normality indicated that most study variables were not normally distributed, except for attention bias (see Supplementary Material for full details). Therefore, for the main analyses, a set of non-parametric Spearman’s bivariate correlations between cognitive biases (at T1) and symptomatology (at T2) and change scores in each type of bullying victimization received from T1 to T2 were first conducted to analyze the temporal relations between these variables. Once the relationships between the variables were verified, the hypothesized structural equation model was then tested including the relationships that were found to be significant. Within the structural equation model, a serial mediation model was tested with negative attention bias at T1 as the exogenous variable, negative interpretation biases at T1 as the first mediator, emotional symptoms (i.e., depression and anxiety) at T2 as second mediators, and finally, the change scores for each type of bullying victimization received from T1 to T2 as the outcome variables. In addition to testing this hypothesized model, a reverse model was tested in which cognitive biases (T1) acted as independent variables directly predicting the different types of victimization (T1-T2), and these, in turn, predicted the subsequent levels of emotional symptoms (T2) (see Supplementary Material for full details).

The model was fitted using maximum likelihood estimation (FIML). For the assessment of the general model fit, the χ² test, the root mean squared error of approximation (RMSEA), the standardized root mean square (SRMR), as well as the confirmatory fit index (CFI) and the Tucker-Lewis index (TLI) were evaluated as comparative fit indices. The following criteria for a well-fitted model (Ruiz et al., 2010) were used: (a) χ² test statistic not significant (*p* >.05); (b) χ²/df value < 3; b) RMSEA value ≤ 0.08; (c) SRMR value close to 0; (d) CFI value ≥ 0.95; (e) TLI value ≥ 0.95. Finally, the mediation effects hypothesized in the path model were tested by estimating indirect effects.

## Results

### Preliminary Analyses

Before starting the data analysis, a simple imputation of missing values was performed to avoid further reducing the original sample and statistical power. The sample had 1.4% missing values and 12.8% cases in psychological variables. Little’s MCAR test was not significant (χ²(88) = 82.6, *p* =.64), so missing values were considered as missing completely at random. Since a minimal amount of values (1.4%) were missing completely at random, a simple Expectation-Maximization (EM) imputation was performed. After imputing missing values, a sensitivity analysis compared the means obtained from the original sample and the sample with imputed data. No significant differences were found (see Supplementary Material).

Additionally, to address potential bias due to task-related attrition in the attention bias task (completed by 62% of the sample), we compared completers and non-completers on all study variables. No significant differences were found, and both groups had identical follow-up rates at T2 (99.07%), suggesting the subsample was representative (see Supplementary Material).

The descriptive statistics for all the resulting study variables are presented in Table 1.

**Table 1:** Mean and standard deviation

		T1		T2	
	Range	Mean	Standard Deviation	Mean	Standard Deviation
Age	13–16	14.54	0.70	-	-
Sex	1–2	1.46	0.50	-	-
Nationality	1–2	1.06	0.23	-	-
CMIE-IV Physical Victimization	5–20	6.58	1.82	5.94	1.54
CMIE-IV Verbal/Relational Victimization	8–32	12.74	3.77	11.47	3.76
CMIE-IV Cyberbullying Victimization	5–20	6.05	1.56	5.69	1.33
SMFQ (depressive symptoms)	0–26	8.59	6.51	8.04	7.26
GAD (anxiety symptoms)	0–21	7.74	5.65	7.31	6.01
Attention Bias	0–1	0.48	0.05	-	-
Interpretation Bias	0–1	0.32	0.24	-	-

### Associations among Cognitive Biases, Symptomatology and Peer Victimization Received

As mentioned above, normality assumptions for most variables were not met (see Supplementary Material for full details). Thus, Spearman correlations were conducted. Results showed a significant positive association between negative attention and interpretation biases at T1 (*ρ* = 0.27, *p* <.001). More importantly, negative interpretation bias at T1 was positively related to higher levels of depression and anxiety symptomatology at T2 (three months later) - (depression: *ρ* = 0.41, *p* <.001; anxiety: *ρ* = 0.39, *p* <.001). Additionally, depression symptomatology at T2 was positively associated with changes from T1 to T2 in the different types of peer victimization (physical: *ρ* = 0.19, *p* =.01; verbal-relational: *ρ* = 0.25, *p* <.001; cyberbullying: *ρ* = 0.18, *p* =.01). That is, higher levels of depression at Time 2 were associated with higher maintenance of peer-victimization experiences from T1 to T2. Anxiety symptoms at T2 were associated only with changes in peer verbal-relational victimization from T1 to T2 (*ρ* = 0.21, *p <*.01), and cyberbullying victimization (*ρ* = 0.16, *p* <.05) (See Table 2).

**Table 2:** Correlations between the study variables

	1	2	3	4	5	6	7
1. Attention Bias (T1)	1						
2. Interpretation Bias (T1)	.27***	1					
3. Depressive symptoms (T2)	.13	.41***	1				
4. Anxiety symptoms (T2)	.17*	.39***	.85***	1			
5. Physical Victimization (Standard Residual)	–.01	.05	.19*	.11	1		
6. Verbal-relational Victimization (Standard Residual)	–.11	–.01	.25***	.21**	.58***	1	
7. Cyberbullying Victimization (Standard Residual)	.04	.01	.18*	.16*	.31***	.43***	1

*p <.05; **p <.01; ***p <.001

### Structural Equation Modeling

**Fig. 3:**
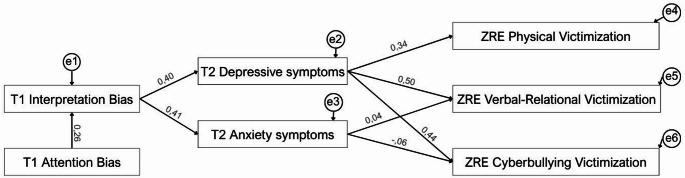
The initial hypothesized model with standardized regression weights. Note. ZRE = Standardized residuals indicating changes from T1 to T2 in each form of peer victimization

In accordance with theoretical models and the results of the bivariate correlations between variables, a structural equation model was tested. Cognitive biases at T1 directly predicted depressive and anxious symptomatology at T2. Additionally, through symptomatology, cognitive biases indirectly predicted changes in the different types of peer victimization from T1 to T2 (see Fig. 3).

**Fig. 4:**
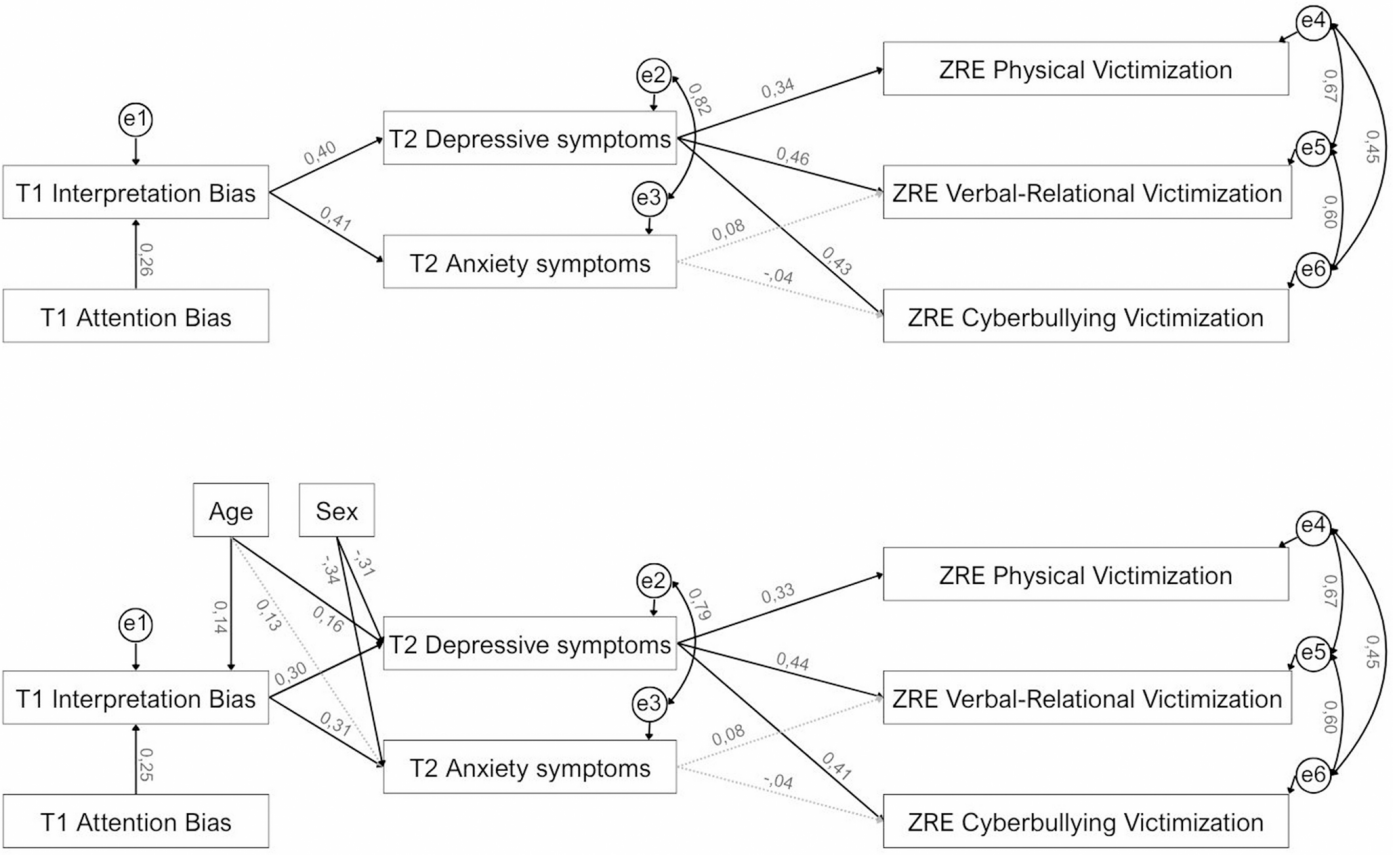
The fitted path models 1R and 1RC with standardized regression weights. Note The discontinuous lines show the non-significant relationships in the model (*p* >.05). ZRE_ Standardized residuals indicating changes from T1 to T2 in each form of peer victimization

As can be seen in Table 3, the initial hypothesized model (Model 1) showed poor fitting, so rectifications were carried out following the Wald and Lagrange multiplier tests (Kline, 2015). The trajectories of the final rectified model (Fig. 4) showed a very good fit for all indices (Model 1R in Table 3). Furthermore, the multivariate normality of the model was supported, as indicated by a Mardia’s coefficient of 9.77 (i.e., < 70; see Rodríguez & Ruiz, 2008). In this final model, anxiety symptoms did not show a direct effect on peer victimization, but it was decided not to remove the variable from the model due to its informative value and covariance with undergoing levels of depressive symptoms, which remained as a significant factor associated with changes in all forms of peer victimization. Also, the reverse model (Model 2) where both forms of symptomatology were included as outcome variables and changes in peer victimization as mediators was tested. The fit of this alternative model was poor (see Supplementary Material), further supporting the results of the final model 1R presented (Fig. 4). Finally, because preliminary analyses identified effects of demographic variables in some of the main variables of the supported 1R Model, they were further controlled in the equation. Specifically, there were differences in sex, with girls showing higher depressive and anxiety symptoms than boys (Chi square = 56.85, p=. 001; and Chi square = 46.15, p=. 002, respectively). Also, age was positive correlated with higher interpretation bias, depressive symptoms, and anxiety symptoms (*ρ* = 0.16, *p* <.05; *ρ* = 0.20, *p* <.01; *ρ* = 0.18, *p* <.05, respectively). Thus, a final model 1RC (i.e., revised while controlling for demographic covariates), was also studied, showing the satisfactory goodness of fit observed in the Model 1R.

**Table 3:** Goodness-of-fit indices of the tested models

Model	Chi-square (df)	*P* value	χ²/df	CFI	TLI	RMSEA (90% CI)	AIC
1	387.7 (13)	< 0.001	29.8	0.32	− 0.09	0.40 (0.3 − 0.44)	431.8
2	446.5 (12)	< 0.001	37.2	0.22	− 0.37	0.45 (0.42-0.49)	492.5
1R	3.9 (9)	0.92	0.43	1.00	1.02	0.00 (0.00-0.03)	55.9
1RC	26.7 (19)	0.11	1.40	0.99	0.98	0.05 (0.00 − 0.09)	96.7

Note. CFI: comparative fit index; TLI: Tucker-Lewis index; RMSEA: root mean square error of approximation; AIC: Akaike; information criterion; Model 1: initial model; Model 2: alternative model; Model 1R: initial model respecified; Model 1RC: respecified model including covariates

Indirect effects of the final model 1RC were tested using bias-corrected Bootstrap estimation (2000 bootstrap samples with 95% CI). Table 4 shows the indirect effects between negative attention bias at T1 and changes from T1 to T2 in different types of peer victimization via interpretation bias at T1 and depressive symptoms at T2. Results showed that the three indirect effects within the model were statistically significant. This supports a pathway in which more negative attention biases led to higher levels of negative interpretation bias at T1. In turn, this predicted higher depressive symptom levels at T2, which then accounted for higher maintenance in the three forms of peer victimization from T1 to T2.

**Table 4:** Bootstrap mediational analysis (Model 1RC)

Variable	Indirect effects (95% CI)		SE	*P* value
Indirect effects via interpretation bias and depression	Lower	Upper		
Attention bias -> physical peer victimization	.31	1.41	.78	<.001
Attention bias -> verbal-relational peer victimization	.98	4.56	2.52	<.001
Attention -> cyberbullying victimization	.28	1.61	.84	<.001

## Discussion

This study examined whether cognitive biases (negative attention and interpretation biases) predict temporal changes in different types of peer victimization and whether this relationship is mediated by emotional symptomatology (symptoms of depression and anxiety). This is an important add on to existing literature, since it has been previously established that the occurrence of emotional symptomatology may aggravate peer victimization experiences in adolescents (Christina et al., 2021). Understanding the role of cognitive mechanisms preceding the appearance of emotional problems may thus be essential for the early identification of adolescents at potential risk of suffering from emotional problems and related victimization processes.

The study tested four different hypotheses. According to our first hypothesis, we expected that negative attention biases would predict negative interpretation biases. Our results fully support this hypothesis, showing that those adolescents who reported higher scores of negative attention biases also reported higher negative interpretation biases at T1. This is in line with previous theories (i.e., Combined Cognitive Bias Hypothesis, Everaert 2012) and previous empirical evidence in adolescents (Sfärlea et al., 2021). Regarding our second hypothesis, we expected to find a direct effect of cognitive biases at T1 on depressive and anxious symptomatology at T2, in line with previous cognitive theories (Beck, 1976; Williams et al., 1997) and recent meta-analyses on the study of these variables in adolescents (Platt et al., 2017; Stuijfzand et al., 2018). Results partially supported this second hypothesis. First, results did support a positive association between negative interpretation bias at T1 and subsequent levels of depressive and anxious symptoms at T2. Yet, these results are in accordance with previous research showing a positive relation between negative interpretation biases and emotional outcomes in adolescents (e.g. Orchard & Reynolds, 2018, Osinsky et al., 2012; Klein et al., 2018; Smith et al., 2018). Our results did not support the same hypothesized pattern of association between negative attention biases and emotional symptomatology. However, this aligns with current theories that have proposed that the effects of attention biases on emotional symptoms may rely on its influence in subsequent stages of the emotional processing (i.e., biases in the interpretation of the information gathered by the attentional system) (see Everaert et al., 2014). In fact, our structural equation model supported this association, where higher negative interpretation biases acted as a mediator in the relationship between higher negative attention biases and subsequent higher emotional symptoms.

Regarding our third hypothesis, we expected to find a direct effect of depressive and anxiety symptomatology at T2 on accounting for the temporal changes from T1 to T2 in peer victimization experiences (i.e., for each type of peer victimization: physical, verbal-relational and cyberbullying). Results of the correlation analyses supported this idea for both depressive and anxiety symptoms, since the presence of higher levels of emotional symptoms at T2 was related to maintenance in all the different peer victimization processes from T1 to T2. However, our results are different from those obtained by Forbes et al. (2019) where a specificity effect emerged (i.e., depression was related to physical and verbal victimization, whereas anxiety was related to cyberbullying victimization). In this sense, our results suggest that emotional symptoms were similarly predictive of changes in physical, verbal-relational, and cyberbullying victimization when assessed across a three-month follow-up. It is worth to noting that in Forbes et al. (2019), participants were assessed on the experience of bullying experiences one year after assessing emotional symptomatology, while, in the present study, we use a shorter temporal window of follow-up of three months. It might be possible that such specific effects observed by Forbes et al. (2019) could be only revealed over the long-term. This highlights the interest in conducting multiple follow-ups to study the contribution of emotional symptomatology and their related mechanisms to different forms of peer victimization both in the mid- and long-term in future research.

Finally, the most novel and interesting result in our study was the support for a structural equation model where cognitive mechanisms (i.e., negative attention and interpretation biases) at T1 predicted maintained forms of physical, verbal-relational and cyberbullying peer victimization from T1 to T2 through their influence in subsequent emotional symptomatology levels at T2. Interestingly, our model revealed that only depressive symptoms, but not anxiety symptoms, mediated of these temporal relationships. A plausible explanation for these results is emotional manifestations of anxiety symptoms (e.g., worry, nervousness) are more common and normalized within the academic context (Jiménez-Mijangos et al., 2023a). Consequently, these manifestations may not be interpreted as a sign of vulnerability in this context (i.e., being less consistently related with changes in peer victimization processes in the mid-term). Also, it is important to note that in our study, depressive and anxiety symptoms showed high covariance, which may reduce the model’s ability to demonstrate direct or specific effects when controlling for their covariance. In addition, the present study’s findings contrast with those reported by Forbes et al. (2019), who found that anxiety predicted cyberbullying victimization. Notably, our study employed a self-reported anxiety measure, whereas the Forbes et al. (2019) study utilized a multi-report questionnaire administered by teachers and family members. This discrepancy in methodology may be a contributing factor to the observed differences in results between the two studies. Future studies should take this into account. In any case, the results support a greater specificity of depressive symptoms as a mediator in the relationship between cognitive biases and temporal changes in peer victimization over three months. These results have relevant implications for preventing peer victimization within the context of bullying. For example, the experimental task used for the assessment of cognitive biases has also been adapted for use in cognitive bias modification trainings. These adaptions have shown promising results in reducing depressive symptoms by targeting negative attention and interpretation biases in adults (Blanco et al., 2023). Future research should extend and replicate our novel findings, as they may inform the development of new tools for the early detection and prevention of emotional disturbances and peer victimization risk in adolescents. Such tools could focus on assessing and modifying negative cognitive biases.

Despite the relevance of these findings, this study has several limitations. Regarding the assessment of peer victimization, it should be noted that we used a rather general questionnaire assessing peer victimization (CMIE-IV; Caballo et al., 2012). Despite our efforts to categorize the items into subscales representing different types of peer victimization and to expand the information on bullying with new representative items, the psychometric properties of the physical and cyberbullying victimization subscales could be improved. Specifically, the alpha coefficients of physical and cyberbullying victimization were found to be questionable, which may affect the reliability of these two measures. Therefore, these results should be interpreted with caution. Future studies aiming to replicate these findings should consider using more specific instruments to assess different types of peer victimization. Next, completing the self-reported questionnaires to assess emotional symptoms and peer victimization using a paper-and-pencil format resulted in some missing data. Although the missing values were completely random, they affected 12.8% of cases when estimating psychological variables. While expectation-maximization (EM) imputation was used to minimize sample loss, future studies should consider using digital tools to reduce the likelihood of missing data during collection. Finally, although this longitudinal study offers several strengths for examining temporal relationships between the variables, it included only two assessments points. It would have been interesting to include a third longer measurement time allowing us to track more long-term changes in peer victimization. For example, in the Forbes et al. (2019) study —with a one-year follow-up— anxiety symptoms predicted later cyberbullying victimization. Thus, including multiple follow-ups might allow us to identify different relationships among different types of emotional symptoms and peer victimization across time. This would also extend the support of our model when negative cognitive biases are considered predictors of changes in these variables at different mid- and long-term periods.

Despite the limitations noted above, this study has several noteworthy strengths. One main strength is the use of a longitudinal design in a sample of adolescents examining variables such as cognitive biases, which have not been studied in the context of peer victimization. To our knowledge, this is the first study that has longitudinally tested the role of cognitive biases and emotional symptoms in predicting perceived peer victimization over time. In addition, including change variables allowed us to study temporal trajectories in different types of peer victimization. Moreover, the current study used the SST, a highly reliable measure of cognitive biases, which further strengthened the robustness of our findings. Finally, our results highlight the central role of cognitive biases in understanding the emergence of depressive symptomatology and its impact on adolescent peer victimization. As noted above, this finding has important clinical implications for preventing emotional problems and peer victimization in adolescents. It suggests that preventive programs targeting cognitive biases may help reduce peer victimization by first reducing emotional symptoms. In conclusion, this study provides valuable empirical evidence supporting the link between negative cognitive biases of attention and interpretation and peer victimization through depressive symptomatology. These results reinforce the evidence for the role of depressive symptomatology as a predictor of peer victimization and highlight the relationship between attention and interpretation biases in adolescents. Overall, this research contributes to the literature on preventing peer victimization and depressive symptoms in adolescents and underscores the key role of cognitive biases in shaping the interconnected emotional and relational processes that unfold during this critical development period.

## Electronic Supplementary Material

Below is the link to the electronic supplementary material.

Supplementary Material 1

## Data Availability

The data will be available upon request.
