# Supplementary material for: Cognitive Biases and Emotional Symptomatology as Predictors of Changes in Peer Victimization: A Longitudinal Structural Equation Modeling Study
Source: Res Child Adolesc Psychopathol. 2025 Jun 2;53(9):1325–37. doi: 10.1007/s10802-025-01337-7 (PMC12423155; doi:10.1007/s10802-025-01337-7)
Supplement: Supplementary file 1 — Supplementary Material 1 [file 10802_2025_1337_MOESM1_ESM.docx]

**Cognitive Biases and Emotional Symptomatology as Predictors** **of Changes in Peer Victimization: A Longitudinal Structural Equation Modeling Study**

**Supplementary Material**

*New items added to the Victimization Received Factor (CMIE-IV)*

| **Physical Bullying** |
| --- |
| 1. *My belongings have been taken, damaged, or stolen without my permission. [Me han cogido, roto o robado cosas sin mi permiso].* |
| **Verbal-Relational Bullying** |
| 1. *They have tried to turn my classmates against me* *[Han tratado de poner a mis compañeros en mi contra].* |
| **Cyberbullying** |
| 1. *They ignore me (ignore my messages, friend requests, etc.) via mobile and internet (WhatsApp, TikTok, Twitter, Instagram...) [Me hacen el vacío (ignoran mis mensajes, solicitudes de amistad, etc) a través del móvil e internet (WhatsApp, TikTok, Twitter, Instagram…)].* 2. *They have shared sexual images or videos of me on the internet or passed them to them via their mobile phone without my consent [Han compartido imágenes o vídeos míos de contenido sexual en internet o se los han pasado a través del móvil sin mi consentimiento].* |

Confirmatory factor analyses (CFA) were conducted using the robust weighted least squares weighted least squares estimator of mean and variance adjusted (WLSMV) for each type of peer victimization (CMIE-IV; Caballo et al., 2012) including ad-hoc items. The results for each model are shown below:

|  |  | χ²(df) | *P* | CFI | TLI | RMSEA (90% CI) | SRMR |
| --- | --- | --- | --- | --- | --- | --- | --- |
| Physical | T1 | 3.56 (5) | 0.61 | 1.00 | 1.01 | 0.00 (0.00-0.09) | 0.07 |
|  | T2 | 6.60 (5) | 0.25 | 0.99 | 0.99 | 0.04 (0.00-0.12) | 0.09 |
| Verbal-Relational | T1 | 19.64 (20) | 0.48 | 1.00 | 1.00 | 0.00 (0.00-0.06) | 0.09 |
|  | T2 | 26.43 (20) | 0.15 | 0.99 | 0.99 | 0.04 (0.00-0.08) | 0.06 |
| Cyber | T1 | 2.51 (5) | 0.77 | 1.00 | 1.01 | 0.00 (0.00-0.07) | 0.04 |
|  | T2 | 3.09 (5) | 0.68 | 1.00 | 1.01 | 0.00 (0.00-0.08) | 0.17 |

Note. CFI: comparative fit index; TLI: Tucker-Lewis index; RMSEA: root mean square error of approximation; SRMR: Standardized Root Mean Residual.

**Attrition analyses**

*Comparison of Participants Who Completed vs. Did Not Complete the Attention Bias Task on Study Variables*

|  | Time | AB complete  *M* (*SD*) | AB non-complete  *M* (*SD*) | *t* (df) | *p* | *d* (Cohen) |
| --- | --- | --- | --- | --- | --- | --- |
| Age | 1 | 14.5 (.7) | 14.4 (.6) | -1.2 (289) | .58 | .67 |
| Sex | 1 | 1.48 (.5) | 1.52 (.5) | .55 (289) | .24 | .50 |
| Nationality | 1 | 1.06 (.25) | 1.07 (.2) | .05 (289) | .96 | .25 |
| Physical Victimization | 1 | 6.6 (1.8) | 6.9 (2.5) | -1.23 (281) | .11 | 1.98 |
|  | 2 | 5.9 (1.6) | 5.6 (1.8) | 1.51 (283) | .07 | 1.64 |
| Verbal/Relational Victimization | 1 | 14.8 (4.0) | 15.1 (5.5) | -.41 (280) | .34 | 4.37 |
|  | 2 | 13.4 (3.9) | 12.5 (3.5) | 1.58 (281) | .06 | 3.90 |
| Cyberbullying Victimization | 1 | 6.1 (1.6) | 6.3 (2.0) | -.74 (286) | .23 | 1.72 |
|  | 2 | 5.6 (1.2) | 5.3 (.8) | 1.61 (287) | .05 | 1.18 |
| Depressive symptoms | 1 | 8.1 (6.3) | 9.3 (6.5) | -1.07 (277) | .14 | 6.35 |
|  | 2 | 7.6 (7.0) | 6.6 (6.2) | .99 (281) | .16 | 6.85 |
| Anxiety symptoms | 1 | 7.4 (5.5) | 7.3 (5.7) | .10 (282) | .46 | 5.58 |
|  | 2 | 6.9 (5.9) | 5.6 (4.9) | 1.46 (286) | .07 | 5.71 |
| Attention Bias | 1 | .48 (.06) | - | - | - | - |
| Interpretation Bias | 1 | .33 (.28) | - | - | - | - |

**p*< .05; ***p*< .01; ****p*< .001. AB: Attention Bias.

**Further analyses on missing data, and normality distributions**

*Correlations between the study variables before imputation*

|  | 1 | 2 | 3 | 4 | 5 | 6 | 7 |
| --- | --- | --- | --- | --- | --- | --- | --- |
| 1.Attention Bias (T1) | 1 |  |  |  |  |  |  |
| 2.Interpretation Bias (T1) | .28*** | 1 |  |  |  |  |  |
| 3.Depressive symptoms (T2) | .13 | .40*** | 1 |  |  |  |  |
| 4.Anxiety symptoms (T2) | .18* | .39*** | .87*** | 1 |  |  |  |
| 5.Physical bullying (Standard Residual) | –.02 | .07 | .17* | .13 | 1 |  |  |
| 6.Verbal-relational bullying (Standard Residual) | –.09 | –.02 | .27*** | .24** | .58*** | 1 |  |
| 7.Cyberbullying (Standard Residual) | –.04 | .01 | .19* | .17* | .36*** | .43*** | 1 |

**p*< .05; ***p*< .01; ****p*< .001

*Assessment of Univariate Normality for Study Variables*

|  | Skewness (Std. error) | Kurtosis (Std. error) |
| --- | --- | --- |
| Attention bias (T1) | .06 (.18) | .61 (.36) |
| Interpretation bias (T1) | .60 (.18) | -.25 (.36) |
| Depressive Symptoms (T2) | .77 (.18) | -.59 (.36) |
| Anxiety Symptoms (T2) | .53 (.18) | -.65 (.36) |
| Physical Victimization (Standard Residual) | 1.29 (.18) | 1.96 (.36) |
| Verbal-Relational Victimization (Standard Residual) | .94 (.18) | 1.2 (.36) |
| Cyberbullying Victimization (Standard Residual) | 1.69 (.18) | 4.15 (.36) |

*Assessment of Normality for Model’s Variables*

|  | Skew | c.r. | kurtosis | c.r. |
| --- | --- | --- | --- | --- |
| Attention bias (T1) | .61 | .34 | .56 | 1.52 |
| Interpretation bias (T1) | .59 | 3.26 | -.28 | -.77 |
| Depressive Symptoms (T2) | .77 | 4.19 | -.61 | -1.68 |
| Anxiety Symptoms (T2) | .53 | 2.88 | -.67 | -1.84 |
| Physical Victimization (T2) | 1.80 | 9.85 | 2.55 | 6.96 |
| Verbal-Relational Victimization (T2) | 1.43 | 7.83 | 1.82 | 4.96 |
| Cyberbullying Victimization (T2) | 2.53 | 13.84 | 6.54 | 17.87 |
| Multivariate |  |  | 16.39 | 9.77 |

**Alternative structural equation model**

*Reverse or alternative model (Model 2 in Table 3) with standardized regression weights*


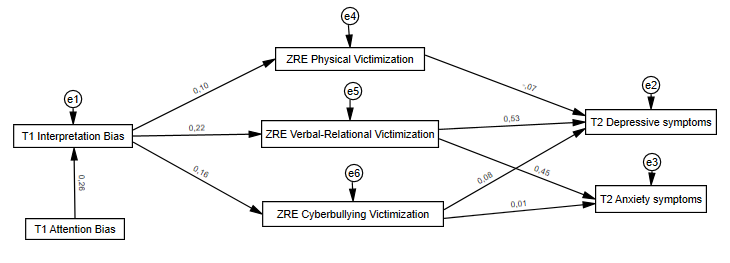


Preliminary Analyses Related to Model 1RC: Chi-Square Tests and Correlations:

*Chi-Square Analysis of Nationality*

|  | χ² | gl | *P* |
| --- | --- | --- | --- |
| Nationality * Attention bias (T1) | 179.00 | 178 | .46 |
| Nationality * Interpretation bias (T1) | 19.58 | 26 | .81 |
| Nationality * Depressive Symptoms (T2) | 13.80 | 27 | .98 |
| Nationality * Anxiety Symptoms (T2) | 24.74 | 22 | .31 |
| Nationality * Physical Victimization (Standard Residual) | 43.49 | 46 | .58 |
| Nationality * Verbal-Relational Victimization (Standard Residual) | 82.94 | 89 | .66 |
| Nationality * Cyberbullying Victimization (Standard Residual) | 16.67 | 33 | .99 |

**p*< .05; ***p*< .01; ****p*< .001

*Chi-Square Analysis of Sex*

|  | χ² | gl | *P* |
| --- | --- | --- | --- |
| Sex * Attention bias (T1) | 179.00 | 178 | .46 |
| Sex * Interpretation bias (T1) | 30.09 | 26 | .26 |
| Sex * Depressive Symptoms (T2) | 56.85 | 27 | .001*** |
| Sex * Anxiety Symptoms (T2) | 46.15 | 22 | .002*** |
| Sex * Physical Victimization (Standard Residual) | 43.63 | 46 | .57 |
| Sex * Verbal-Relational Victimization (Standard Residual) | 85.42 | 89 | .59 |
| Sex * Cyberbullying Victimization (Standard Residual) | 38.99 | 33 | .22 |

**p*< .05; ***p*< .01; ****p*< .001

*Spearman Correlations Between Age and the Main Study Variables*

|  | 1 | 2 | 3 | | 4 | | 5 | | 6 | | 7 | | 8 | |  |
| --- | --- | --- | --- | --- | --- | --- | --- | --- | --- | --- | --- | --- | --- | --- | --- |
| 1. Age | 1 |  | |  | |  | |  | |  | |  | |  | |
| 2. Attention Bias (T1) | .03 | 1 | |  | |  | |  | |  | |  | |  | |
| 3. Interpretation Bias (T1) | .16* | .27*** | | 1 | |  | |  | |  | |  | |  | |
| 4. Depressive symptoms (T2) | .20** | .13 | | .41*** | | 1 | |  | |  | |  | |  | |
| 5. Anxiety symptoms (T2) | .18* | .17* | | .39*** | | .85*** | | 1 | |  | |  | |  | |
| 6. Physical Victimization (Standard Residual) | –.13 | –.01 | | .05 | | .19* | | .11 | | 1 | |  | |  | |
| 7. Verbal-relational Victimization (Standard Residual) | –.10 | –.11 | | –.01 | | .25*** | | .21** | | .58*** | | 1 | |  | |
| 8. Cyberbullying Victimization (Standard Residual) | –.002 | .04 | | .01 | | .18* | | .16* | | .31*** | | .43*** | | 1 | |

**p*< .05; ***p*< .01; ****p*< .001
